# Blood tryptase and thymic stromal lymphopoietin levels predict the risk of exacerbation in severe asthma

**DOI:** 10.1038/s41598-021-86179-1

**Published:** 2021-04-19

**Authors:** Hsin-Kuo Ko, Shih-Lung Cheng, Ching-Hsiung Lin, Sheng-Hao Lin, Yi-Han Hsiao, Kang-Cheng Su, Chong-Jen Yu, Hao-Chien Wang, Chau-Chyun Sheu, Kuo-Chin Chiu, Diahn-Warng Perng

**Affiliations:** 1 Department of Chest Medicine, Taipei Veterans General Hospital, No. 201, Sec. 2, Shih-Pai Road, Taipei, 112 Taiwan, ROC; 2 School of Medicine, National Yang-Ming University, Taipei, Taiwan, ROC; 3 Department of Internal Medicine, Far Eastern Memorial Hospital, New Taipei City, Taiwan, ROC; 4 Department of Chemical Engineering and Materials Science, Yuan Ze University, Zhongli, Taoyuan Taiwan, ROC; 5 Division of Chest Medicine, Changhua Christian Hospital, Changhua, Taiwan, ROC; 6 College of Health Sciences, Chang Jung Christian University, Tainan, Taiwan, ROC; 7 Department of Internal Medicine, National Taiwan University Hospital, Taipei, Taiwan, ROC; 8 Division of Pulmonary and Critical Care Medicine, Department of Internal Medicine, Kaohsiung Medical University Hospital, Kaohsiung, Taiwan, ROC; 9 Department of Internal Medicine, School of Medicine, College of Medicine, Kaohsiung Medical University, Kaohsiung, Taiwan, ROC; 10 Division of Chest, Department of Internal Medicine, Poh-Ai Hospital Luodong, No. 83, Nanchang St., Luodong Township, 265 Yilan Taiwan, ROC

**Keywords:** Biomarkers, Diseases

## Abstract

Some patients with severe asthma experience exacerbations despite receiving multiple therapy. The risk of exacerbation and heterogeneous response to treatment may be associated with specific inflammatory molecules that are responsive or resistant to corticosteroids. We aimed to identify the independent factors predictive for the future risk of exacerbation in patients with severe asthma. In this multi-center prospective observational study, 132 patients with severe asthma were enrolled and divided into exacerbation (n = 52) and non-exacerbation (n = 80) groups on the basis of exacerbation rate after a 1-year follow-up period. We found that previous history of severe-to-serious exacerbation, baseline blood eosinophil counts (≥ 291cells/μL), and serum tryptase (≤ 1448 pg/mL) and thrymic stromal lymphopoietin (TSLP) levels (≥ 25 pg/mL) independently predicted the future development of exacerbation with adjusted odds ratios (AOR) of 3.27, 6.04, 2.53 and 8.67, respectively. Notably, the patients with high blood eosinophil counts and low tryptase levels were likely to have more exacerbations than those with low blood eosinophil counts and high tryptase levels (AOR 16.9). TSLP potentially played the pathogenic role across different asthma phenotypes. TSLP and tryptase levels may be implicated in steroid resistance and responsiveness in the asthma inflammatory process. High blood eosinophil counts and low serum tryptase levels predict a high probability of future asthma exacerbation.

## Introduction

Asthma exacerbation is associated with an increase in respiratory symptoms and progressive decrease in lung function^1^. In patients with severe asthma, 30% of subjects are frequent exacerbators, and these exacerbations impose a huge economic and health burden on health care systems^2–4^. Identifying disease characteristics and selecting effective treatment for patients with severe asthma are important to reduce the future risk of exacerbations^5^. Clinical phenotypes have been described but do not necessarily reflect underlying disease mechanisms^6^. The classification of endotypes have been developed to characterize distinct biological mechanisms, and thus therapy targeting specific molecules could improve disease outcomes in severe asthma^7–10^. Theoretically, endotype-related molecular/cellular biomarkers may be associated with the responsiveness to corticosteroids and could be applied to predict the future risk of exacerbation in patients with severe asthma^9, 10^.

The inflammatory mechanism of asthma is currently divided into type 2 high and type 2 low (non-type 2) inflammatory processes. Type 2 high inflammation includes both allergic and non-allergic eosinophilic processes^8, 10, 11^. In allergic asthma, exposure to an allergen results in the production of interleukin (IL)-4, IL-5, and IL-13 by T helper 2 lymphocyte (T_H_2). The cytokines of IL-4 and IL-13 stimulate B lymphocytes to produce antigen-specific immunoglobulin (Ig) E that drives the allergic cascade. IL-5 can increase the production, differentiation, maturation and activation of eosinophils. In non-allergic eosinophilic asthma, type 2 innate lymphoid cells (ILC2) appear to be responsible for the production of type 2 cytokines IL-5 and IL-13. Accumulating evidence shows that T_H_2 cells and T_H_2-driven eosinophilia are usually responsive to glucocorticoids^12–14^. Clinically, a substantial proportion of patients with asthma does not respond to glucocorticoids very well^15^.

Thymic stromal lymphopoietin (TSLP), which is mainly derived from epithelium, can promote the activation of dendritic cells and B lymphocytes as well as T_H_2-associated cytokine production^16^. TSLP can also induce chemotaxis and delay apoptosis in eosinophils, suggesting its potential role in allergic inflammation^17^. In addition to eosinophilic inflammation, TSLP also plays a role in neutrophilic airway inflammation^18, 19^ and promotes airway remodeling^20–23^. Moreover, TSLP exerts a corticosteroid-resistant effect in natural helper cells by controlling STAT5 phosphorylation and BCL-xL expression^24^. As the major protein component in the mast cells, tryptase has been recognized as a specific marker of mast cell activation and involved in allergic asthma^25^. Serum tryptase can be used to predict disease severity in childhood asthma^26^. In induced sputum, tryptase concentration can be reduced by high doses of inhaled corticosteroid (ICS) within 6 h in symptomatic asthmatics^27^. Targeting the T_H_2 pathway can inhibit late asthmatic response by attenuating allergen-induced sputum eosinophilia and lowering tryptase levels^28^.

Eosinophils are involved in the pathogenesis of asthma exacerbation. Blood eosinophils are reportedly associated with the frequency of asthma exacerbation^29^. A UK cohort study found that asthmatics with blood eosinophil counts higher than 400 cells/μL experience more severe exacerbations and poorer asthma control^30^. A treatment strategy specifically aimed to reduce sputum eosinophilia can decrease asthma exacerbation and hospitalization rate^31^. Collectively, the extent of eosinophilic inflammation appears to be associated with uncontrolled asthma. On the basis of these pieces of evidence, we hypothesized that clinical characteristics and inflammatory biomarkers may simultaneously affect patient outcomes and are independently associated with the future risk of exacerbation in patients with severe asthma.

We conducted a 1-year multicenter prospective observational study aimed to identify the clinical characteristics and useful biomarkers that can independently predict the risk of exacerbation in patients with severe asthma under maintenance treatment. We demonstrated that previous history of exacerbation, blood eosinophil count, and tryptase and TSLP levels can be used as independent factors for predicting future asthma exacerbations.

## Methods

### Study design

This prospective, observational, multi-center study was conducted at six hospitals across Taiwan from March 2016 to February 2018. The study was approved by the Institutional Ethical Review Board of Taipei Veterans General Hospital, Far Eastern Memorial Hospital, National Taiwan University Hospital, Changhua Christian Hospital, Kaohsiung Medical University Hospital and Lotung Poh-Ai Hospital (approval number: VGHTPE-IRB No. 2016–03-010AC) and conducted in accordance with the Declaration of Helsinki. All patients provided written informed consent for participation, and the study was registered at https://www.clinicaltrials.gov (NCT02871947). The patients enrolled were followed-up for 1 year after enrollment.

### Participants

Patients were outpatients aged 20–75 years with at least a 1-year history of asthma and a current diagnosis of severe asthma under GINA steps 4–5 therapy^1^ with a high-dose ICS (≥ 800 μg of budesonide or equivalent) and a long-acting β2 agonist, sustained-release theophylline or leukotriene receptor antagonist for the previous 6 months before enrollment or oral glucocorticosteroids (OCS) were prescribed in stable doses for the previous 3 months^32^. Patients were never smokers or had a smoking history of less than 10 pack-years. The main exclusion criteria were an event of asthma exacerbation treated with systemic glucocorticoids within 4 weeks before enrollment, chronic obstructive pulmonary disease, active malignancy, infectious diseases, active pulmonary tuberculosis, and a current treatment of home oxygen therapy ≥ 15 h per day and noninvasive positive pressure ventilation ≥ 6 h per day.

### Measurements

The demographic information and clinical data including history of exacerbation, current treatment, atopy and comorbidities were collected. At the time of enrollment, the participants were assessed for Asthma Control Test (ACT); bronchodilator test according to the American Thoracic Society criteria^33^; blood cell counts; fractional exhaled nitric oxide (FeNO); blood serum IgE; and associated mediators including interleukin IL-5, IL-13, periostin, tryptase, IL-8, IL-17, tumor growth factor-β, vascular endothelial growth factor, placental growth factor, tumor necrosis factor-α, TSLP, and IL-33. The serum levels of cytokines and mediators were analyzed by ELISA kits with a validation control and Bio-Plex Suspension Array System with a validation kit control (#64080422). Serum tryptase β-2 levels were measured by Human Tryptase/TPSAB1, B2 PicoKine™ ELISA Kit. (Catalog #EK0898, Boster Biological Technology, Pleasanton CA, USA).

### Definitions

Reversibility in the bronchodilator test was defined as an increase of 12% and 200 mL in FEV_1_^1^. Uncontrolled asthma was defined as at least one of the following: (a) poor symptom control: ACT < 20; (b) frequent severe exacerbations: two or more bursts of systemic OCS (> 3 days each) in the previous year; (c) serious exacerbations: at least one hospitalization, intensive care unit stay or mechanical ventilation in the previous year; (d) airflow limitation: after appropriate bronchodilator with forced expiratory volume in one second (FEV_1_) < 80% predicted and FEV_1_/forced vital capacity < 0.7^32^. Severe exacerbation was defined as a worsening of asthma requiring the use of systemic corticosteroids for more than 3 days, whereas serious exacerbation was defined as requiring asthma-specific emergency department visits or hospitalization^32^. Atopic status was defined as the positive result of blood allergen-specific IgE. Previous history of asthma exacerbation was defined as the occurrence of severe or serious asthma within 1 year prior to the study entry. Electronic medical record and clinical information of asthma exacerbations throughout the follow-up period were assessed and recorded every 3 months.

### Statistical analysis

Categorical variables were expressed as number (percentage) and evaluated by Chi-square test. Continuous variables with normal distribution were expressed as mean ± standard deviation (SD) and evaluated by independent t-test. Continuous variables with non-normal distribution were expressed as median (interquartile range) and evaluated by Mann–Whitney U test. Variables significantly associated with asthma exacerbation (*P* < 0.05) on univariate analysis were included in multivariate logistic regression analysis. Correlation between two variables was tested by Spearman’s correlation analysis. The enter method was employed to identify the significant predictors. ROC analyses were performed to obtain area under curves (AUC) and the optimal cut-off values were determined by the largest values of Youden’s index with reliable sensitivity, specificity, positive predicted value, and negative predicted value for predicting asthma exacerbation. Finally, Kaplan–Meier survival curves were compared using the log-rank test to analyze the difference in time to severe-to-serious exacerbation between the study patients with and without an independent predictive factor. The power (1 − β) of the sample size was evaluated by G-power program. Results were considered significant at *P* < 0.05 and all p values were two-sided. Statistical analysis was performed using SPSS version 19.0 (SPSS Inc., Chicago, IL, USA).

## Results

### Demographic characteristics of study subjects

A total of 132 study subjects who fulfilled the criteria of severe asthma, including 82 females and 50 males with a median age of 62.5 (55.0–72.0) years, were recruited (Table 1). The median duration of asthma diagnosis was 5.0 (2.0–12.0) years. Among these participants, 29% had smoking history and 36% had a family history of asthma. Moreover, 55% of the study patients were atopic, and the median serum IgE level and blood eosinophil count were 101.0 IU/mL (30.1–320.0) and 187.6 cells/μL (84.0–365.7), respectively. The most common comorbidities were allergic rhinitis, hypertension, and diabetes mellitus. The median baseline prebronchodilator FEV1 and FEV1% pred were 1.45L (0.96–2.05) and 65.3% (50.3–80.7), respectively, and 52% of the participants had fixed airflow limitation. Only 15% of the study subjects had positive bronchodilator reversibility and 7% of the patients received maintenance oral corticosteroids. The median score of ACT was 21 (19–23), 70% of which were defined as uncontrolled asthma at the commencement of the study.

**Table 1:** Baseline characteristics of the study subjects (n = 132).

	Total (n = 132)	Exacerbation (n = 52)	Non-exacerbation (n = 80)	*P*
Female (%)	82 (62)	33 (63)	49 (61)	0.855
Age (year)	62.5 (55.0–72.0)	60.5 (56.4–63.4)	63.5 (59.5–65.5)	0.166
Duration of asthma diagnosis (year)	5.0 (2.0–12.0)	7.0 (3.0–16.0)	5.0 (2.0–12.3)	0.293
Smoking hx (%)	39 (29)	16 (31)	23 (29)	0.847
Current smoker (%)	5 (4)	1 (2)	4 (5)	0.648
Body mass index (kg/m^2^)	24.6 (22.0–27.7)	24.6 (22.2–26.9)	24.4 (21.3–26.7)	0.310
Family history of asthma (%)	48 (36)	21 (40)	27 (34)	0.464
Atopy (%)	72 (55)	24 (46)	48 (60)	0.153
**Comorbidity**				
Allergic rhinitis (%)	95 (72)	35 (67)	60 (75)	0.428
Hypertension (%)	60 (45)	24 (46)	36 (45)	1.000
Diabetes mellitus (%)	27 (20)	9 (17)	18 (23)	0.515
GERD (%)	23 (17)	9 (17)	14 (18)	1.000
Heart failure (%)	12 (9)	5 (10)	7 (9)	1.000
Bronchiectasis (%)	6 (5)	3 (6)	3 (4)	0.680
Nasal polyp (%)	4 (3)	2 (4)	2 (3)	0.646
**Pulmonary function test**				
FEV1/FVC (%)	66.9 (56.8–76.8)	66.4 (59.2–74.8)	66.6 (54.8–76.9)	0.845
FEV1 (liter)	1.45 (0.96–2.05)	1.54 (0.96–2.06)	1.40 (0.99–2.09)	0.970
FEV1%pred (%)	65.3 (50.3–80.7)	68.0 (46.3–80.6)	63.0 (50.4–80.3)	0.543
FVC (liter)	2.14 (1.59–2.91)	2.20 (1.59–2.94)	2.17 (1.61–2.96)	0.944
FVC %pred (%)	79.6 (66.4–94.8)	78.0 (61.0–95.0)	80.1 (69.4–106.9)	0.495
Positive BDR (%)	20 (15)	7 (13)	13 (16)	0.805
%Reversibility of FEV1	5.0 (1.0–9.9)	4.5 (0.2–8.4)	5.0 (1.0–11.2)	0.228
Fixed airflow limitation (%)	68 (52)	27 (52)	41 (51)	1.000
**Use of ICS and**				
LABA (%)	91 (69)	33 (63)	58 (73)	0.336
LABA and LAMA (%)	31 (23)	11 (21)	20 (25)	0.678
Leukotriene modifier (%)	56 (42)	19 (37)	37 (46)	0.286
Theophylline (%)	80 (61)	29 (56)	51 (64)	0.369
Anti-histamine (%)	8 (6)	2 (4)	6 (8)	0.479
Maintenance oral prednisolone (%)	9 (7)	2 (4)	7 (9)	0.482
Omalizumab (%)	10 (8)	4 (8)	6 (8)	1.000
ACT	21 (19–23)	20.0 (18.7–23.0)	21.0 (18.7–23.0)	0.157
Uncontrolled asthma (%)	92 (70)	39 (74)	53 (66)	0.335
Asthma exacerbation in the year prior to the study (%)	73 (55)	40 (77)	33 (41)	< 0.001
Severe exacerbation (/year)	0.57 ± 0.74	0.77 ± 0.75	0.44 ± 0.70	0.003
Serious exacerbation (/year)	0.18 ± 0.57	0.27 ± 0.66	0.13 ± 0.51	0.079
**Asthma exacerbation during 1-year follow-up**				
Severe exacerbation (/year)	0.45 ± 0.98	1.13 ± 1.29	0	
Serious exacerbation (/year)	0.29 ± 1.01	0.71 ± 1.52	0	

Data were reported as mean ± standard deviation, median (interquartile range) or number (%).

*ACT* asthma control test, *BDR* bronchodilator reversibility, *FEV1* forced expiratory volume in one second, *FVC* forced vital capacity, *GERD* gastroesophageal reflux disease, *ICS* inhaled corticosteroid, *LABA* long-acting beta 2 agonist, *LAMA* long-acting muscarinic antagonist.

The study subjects were divided into two groups on the basis of the occurrence of severe-to-serious exacerbation after a 1-year follow-up period (Table 1). The exacerbation group (n = 52) had severe (1.13/year) and serious (0.71/year) exacerbation during the entire follow-up period compared with the non-exacerbation group (n = 80). Apparently, the history of severe exacerbation in the previous year were higher in the exacerbation group than that in the non-exacerbation group (0.77 ± 0.75 vs. 0.44 ± 0.70, respectively; *p* = 0.003).

### Blood biomarkers in both groups

The blood cellular and molecular biomarkers of the study subjects are summarized in Table 2. In terms of counts of blood eosinophils (absolute number ≥ 300 cells/µl and more than 4%) were significantly higher in the exacerbation group than those in the non-exacerbation group. Serum IgE, periostin, IL-5, and IL-13 levels, as well as FeNO, were not statistically different between the two groups. In particular, significantly lower tryptase levels and higher TSLP levels were observed in the exacerbation group (*p* = 0.010 and 0.016, respectively) comparing with those in the non-exacerbation group.

**Table 2:** Blood cellular and molecular biomarkers in the study subjects (n = 132).

	Total (n = 132)	Exacerbation (n = 52)	Non-exacerbation (n = 80)	*P*
**Cellular markers**				
WBC (cells/µl)	7680 (6190–9400)	8110 (6400–9800)	6900 (5945–8550)	0.052
Eosinophil (cells/µl)	187.6 (84.0–365.7)	241.5 (248.5–458.0)	166.7 (148.8–225.9)	0.016
Eos ≥ 150 cells/µl (%)	81 (61)	36 (69)	45 (56)	0.212
Eos ≥ 300 cells/µl (%)	42 (32)	25 (48)	17 (21)	0.004
Eos ≥ 4% (%)	43 (33)	23 (44)	20 (25)	0.036
Neutrophil (%)	58.9 (51.8–66.2)	57.9 (49.1–65.0)	59.7 (52.6–67.1)	0.232
**Molecular markers**				
IgE, IU/ml	101.0 (30.1–320.0)	109.3 (25.5–296.0)	83.2 (41.7–311.0)	0.769
Tryptase beta-2 (pg/ml)	1053.5 (373.9–2403.3)	768.2 (169.2–1732.8)	1725.5 (517.1–3036.5)	0.010
TSLP (pg/ml)	8.0 (3.5–21.1)	16.3 (3.4–32.3)	7.1 (3.3–18.2)	0.016
FeNo (ppb)	26.0 (19.3–43.8)	31.0 (21.0–43.3)	25.8 (18.0–44.7)	0.205
Periostin (pg/ml)	14.3 (9.4–19.8)	16.7 (11.4–22.1)	13.0 (9.1–18.2)	0.206
IL-5 (pg/ml)	2.2 (1.3–3.0)	2.1 (1.1–3.7)	2.3 (1.3–3.0)	0.882
IL-13 (pg/ml)	62.5 (35.3–76.0)	63.8 (33.3–76.1)	63.2 (34.7–77.6)	0.545
IL-33 (pg/ml)	2.9 (1.0–5.0)	3.1 (1.2–5.9)	2.9 (1.3–4.5)	0.615
TNF-α (pg/ml)	2.5 (1.6–3.6)	2.6 (1.3–3.6)	2.4 (1.6–3.4)	0.906
IL-8 (pg/ml)	6.6 (4.4–11.7)	6.8 (4.5–11.6)	6.7 (4.4–13.1)	0.954
IL-17 (pg/ml)	12.6 (9.9–15.6)	12.7 (10.4–15.1)	12.7 (10.1–15.7)	0.909
TGF-β (pg/ml)	24.9 (19.9–33.3)	26.8 (21.9–34.2)	22.7 (19.3–33.3)	0.063
VEGF (pg/ml)	267.0 (157.9–398.2)	281.9 (167.7–400.8)	266.2 (140.3–416.9)	0.698
PIGF (pg/ml)	4.7 (3.2–7.7)	4.4 (3.2-–.7)	5.3 (4.0–9.5)	0.471

Data were reported as median (interquartile range) or number (%).

*EOS* eosinophil, *FeNO* fraction of exhaled nitric oxide, *PIGF* placental growth factor, *TGF* tumor growth factor, *TNF* tumor necrosis factor, *TSLP* thymic stromal lymphopoietin, *VEGF* vascular endothelial growth factor, *WBC* white blood cell.

### Significant factors associated with asthma exacerbation

**Figure 1:**
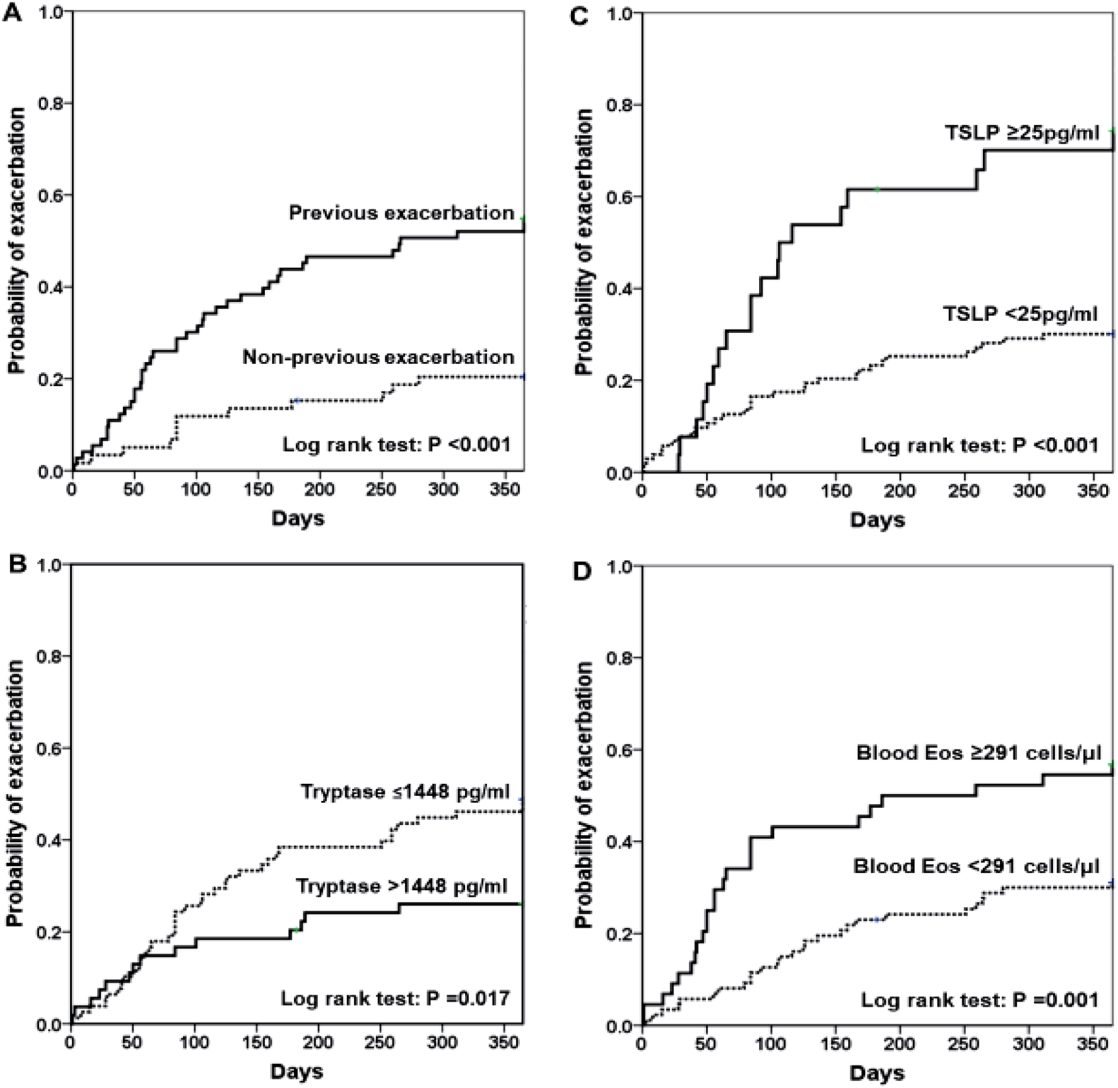
Kaplan–Meier curves of the cumulative probability of exacerbation during the 1-year follow-up period stratified by previous history of asthma exacerbation (**A**), serum tryptase (**B**) and TSLP (**C**) levels, and blood eosinophil count (**D**). The cut-off values of 1448 pg/mL, 25 pg/mL, and 291 cells/µL for serum tryptase level, serum TSLP level, and blood eosinophil count, respectively, were chosen by receiver operating characteristic curve analysis.

The significant factors associated with asthma exacerbation are listed in Table 3. The ROC curve was analyzed for blood eosinophil counts, and serum tryptase and TSLP levels to differentiate the exacerbation group from the non-exacerbation group (Supplementary Fig. 1A–C). The adjusted multivariate logistic regression model revealed that previous history of severe-to-serious asthma exacerbation, serum tryptase level of ≤ 1448 pg/mL, serum TSLP level of ≥ 25 pg/mL and blood eosinophil count of ≥ 291cells/µl, were the independent factors predictive for asthma exacerbation with an AOR of 3.27, 2.53, 8.67 and 6.04, respectively. We analyzed the correlation between blood eosinophil counts, serum tryptase and TSLP levels by Spearman’s correlation analysis, and the results showed that there was no significant correlation between blood eosinophil counts and levels of serum tryptase (r = − 0.059, *p* = 0.516), blood eosinophil counts and levels of serum TSLP (r = 0.109, *p* = 0.222), and levels of serum TSLP and tryptase (r = − 0.166, *p* = 0.065). This finding further confirmed that blood eosinophil counts, and levels of serum tryptase and TSLP as the independent variables for predicting asthma exacerbation. The Kaplan–Meier curves of the cumulative probability of severe-to-serious exacerbation during the 1-year follow-up period stratified by the independent factors are shown in Fig. 1A–D (all log rank test, *p* < 0.05).

**Table 3:** Significant factors associated with asthma exacerbation (n = 132).

Variable	Univariate		Multivariate	
	OR (95%CI)	*P*	AOR (95% CI)	*P*
Previous history of severe-to-serious exacerbation	4.75 (2.17–10.40)	< 0.001	3.27 (1.34–8.00)	0.009
Serum tryptase ≤ 1448 pg/mL	2.47 (1.17–5.19)	0.009	2.53 (1.01–6.36)	0.048
Serum TSLP ≥ 25 pg/mL	6.30 (2.41–16.52)	< 0.001	8.67 (2.63–28.62)	< 0.001
Blood Eos count ≥ 291cells/µl	2.92 (1.38–6.19)	0.005	6.04 (2.30–15.88)	< 0.001

*EOS* eosinophil, *TSLP* thymic stromal lymphopoietin.

### Adjusted odds ratio for future development of asthma exacerbation

**Figure 2:**
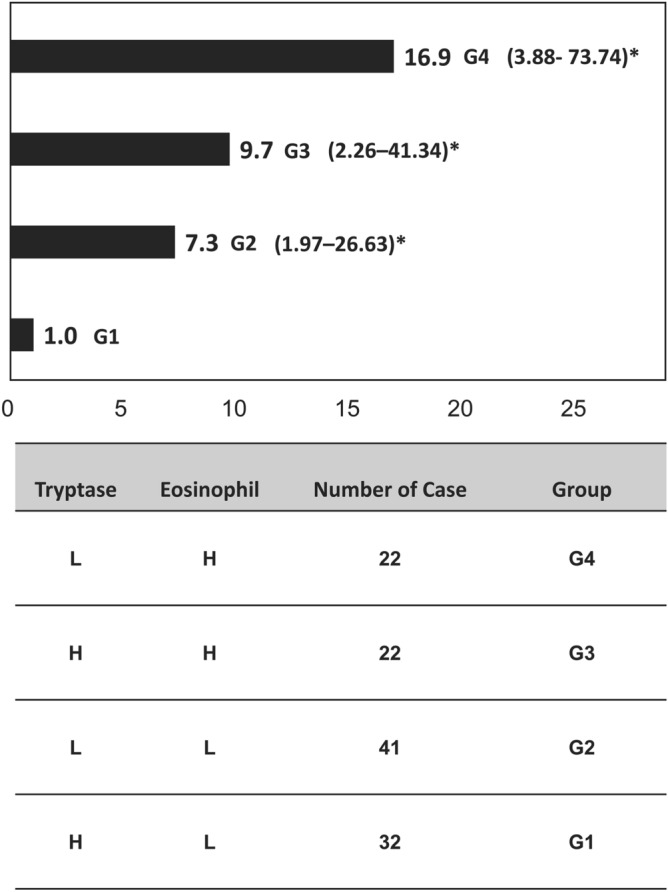
Adjusted odds ratio (AOR) for developing asthma exacerbation during the 1-year follow-up period based on blood tryptase and eosinophil levels. High (H) and low (L) levels of serum tryptase and blood eosinophil count were defined on the basis of the cut-off values of 1448 pg/mL and 291 cells/µL, respectively. * denotes p value < 0.05. When G1 group is defined as reference, the AOR with 95% confidence intervals (95% CI) and p value for asthma exacerbation during the 1-year follow-up period for G4, G3, and G2 are 16.92 (3.88–73.74, *p* < 0.001), 9.67 (2.26–41.34, *p* = 0.002), and 7.25 (1.97–26.63, *p* = 0.003), respectively.

For the analysis of combined biomarkers predictive for the risk of asthma exacerbation, we first categorized the study subjects into 4 groups according to the serum TSLP levels and blood eosinophil counts. Because only 6 cases were grouped in the group of TSLP high/EOS high, we did not choose serum TSLP levels and blood eosinophil counts as combined biomarkers. Alternatively, we chose serum tryptase level and blood eosinophil counts as combined biomarkers. The AOR for future development of asthma exacerbation associated with blood tryptase level and eosinophil count is shown in Fig. 2. Tryptase level of 1448 pg/mL and blood eosinophil count of 291cells/µl were considered as the cutoff. The patients with high eosinophil counts and low tryptase levels were more likely to develop asthma exacerbation than those with low eosinophil counts and high tryptase levels (AOR: 16.92, 95% CI = 3.88–73.74, *p* < 0.001).

## Discussion

Asthma is a heterogeneous disease. Using modeling approaches and cluster analysis, distinct clinical phenotypes of asthma are identified and the clinical characteristics suggest difference in pathophysiologic mechanisms in patients with severe asthma^34^. Biomarker analysis may help in tailoring treatment and predicting the future risk of exacerbation in patients with severe asthma^5, 35, 36^. In this prospective observational study, we demonstrated that previous history of severe-to-serious exacerbation, baseline serum tryptase and TSLP levels, and blood eosinophil counts could independently predict the future development of exacerbation in patients with severe asthma. Most importantly, patients with severe asthma with high blood eosinophil counts and low serum tryptase levels were more likely to have greater risk of exacerbation than those with low blood eosinophil counts and high serum tryptase levels despite treatment with ICS-contained multiple therapy. Our study proposed that the combined biomarkers of serum TSLP and tryptase levels, and blood eosinophil count may be linked to distinct inflammatory mechanisms in asthma and be useful to predict the future risk of asthma exacerbation. The relationship between IL-6 and type2 biomarkers has been investigated by Li et al.^37^, and the authors report that a combination of IL-6 level (representing non-type 2 asthma) and FeNO value or blood eosinophil count (representing type 2 asthma) might identify different asthma endotypes. The findings in the current study strengthened the concept of combined biomarkers being applicable for identifying underling inflammatory endotypes and predicting the future outcomes in patients with severe asthma.

High blood eosinophil count is associated with disease severity and eosinophilic airway inflammation in asthma^38^. In the Copenhagen General Population Study, Vedel-Krogh el al.^39^ reported that increased incidence of moderate-to-severe exacerbation is more strongly associated with high blood eosinophil counts (> 290 cells/μL) than with low blood eosinophil counts (< 180 cells/µl). In the UK, Price et al.^30^reported that patients with asthma with blood eosinophil counts > 400 cells/μL experience more severe exacerbations and have poorer asthma control. Our findings were consistent with these results. Absolute blood eosinophil counts of ≥ 291 cells/μL had a higher probability of asthma exacerbation (AOR = 6.04). In addition, the patients with high blood eosinophil counts and low serum tryptase levels (≤ 1448 pg/mL) simultaneously suffered from more frequent exacerbations than those with low blood eosinophils and high tryptase levels (AOR up to 16.92).

Mast cells are tissue-based inflammatory cells of hematopoietic origin that respond to signals of innate and adaptive immunity. Mast cells play an important role in allergic diseases, including anaphylaxis, allergic rhinitis, and allergic asthma^40^. Human mast cells secrete α- and β-tryptases. Mature β-tryptase, which was measured in this study, is the predominant form stored in the secretory granules of mast cells. Tryptase is a specific marker of mast-cell activation, and thus tryptase levels can be reasonably measured to reflect the burden of mast cell activation in the allergic T_H_2 pathway in asthma^25, 40^. Gao et al.^26^ reported that serum baseline tryptase levels in childhood asthma, as well as asthma control, serum IgE and IL-13 levels, blood eosinophil counts, and lung function parameters, are strongly correlated with disease severity of asthma. The Severe Asthma Research Program also reported that severe asthma is associated with the predominance of tryptase + chymase + mast cells in the airway submucosa and epithelium^41^. In addition, the gene expression of mast cell tryptase is increased in asthmatic epithelium, especially in the T_H_2-high subgroup, and predicts the responsiveness to ICS^42^. The numbers of airway tissue mast cells and the concentration of bronchoalveolar lavage tryptase can determine the efficacy of ICS treatment in persistent asthma^43^. The findings of our study indicating low levels of tryptase associated with a higher risk of exacerbation implied that lower levels of serum tryptase may be linked to non-allergic type 2 inflammation or non-type 2 inflammation (ILC2-related or neutrophilic inflammation). Therefore, the pheno-endotype related to lower levels of serum tryptase is potentially corticosteroid-resistant and refractory to ICS/LABA treatment and associated with high risk of asthma exacerbation^8^.

TSLP, which is produced mainly by the lung and gut epithelia, skin keratinocytes, and dendritic cells, is involved in various allergic diseases, including bronchial asthma, atopic dermatitis, and eosinophilic esophagitis. TSLP release can be triggered by several cytokines, respiratory viruses, bacterial and fungal products, allergens, cigarette smoke extracts, diesel particles and tryptase^44^, and lead to activation of inflammatory responses in asthma^45–48^. Although TSLP is central to type 2 immunity, many cell types that are activated by or respond to TSLP, such as mast cells, basophils, natural killer T cells, ILCs and neutrophils, may play a role in inflammation in asthma beyond type 2 inflammation^47, 49–51^. In asthma, increased TSLP concentrations are observed in bronchoalveolar lavage, induced sputum, exhaled breath condensate, and plasma^52–55^. TSLP expression is increased in the airway mucosa in a subset of severe asthmatics despite high-dose inhaled or oral steroid treatment^56^. TSLP can induce steroid resistance and abrogate the inhibitory effects of dexamethasone on type 2 cytokine production in ILC2 cells^57^. In the present study, we found that TSLP per se is an independent factor for predicting future risk of asthma exacerbation, and serum TSLP levels ≥ 25 pg/mL are associated with a high probability of asthma exacerbation (AOR = 8.19). Unsurprisingly, Corren et al.^58^ reported that anti-TSLP monoclonal antibody reduces annual exacerbation rates by 62%–71% at different doses in uncontrolled asthma despite treatment with long-acting β2 agonists and medium-to-high doses of ICS. Their findings have suggested some biological plausibility for TSLP being a contributor and an indicator of asthma exacerbation, and highlight the potential pathogenic role of TSLP across different asthma phenotypes. Collectively, serum TSLP may contribute to steroid resistance, whereas tryptase may suggest steroid responsiveness in asthma inflammatory process, as observed in the present study. Moreover, our study suggested the novel idea that the possible combination of elevated TSLP levels and reduced tryptase levels might result in ongoing eosinophilia and non-responsiveness to high-dose ICS treatment. This combination of biomarkers (high TSLP levels and low tryptase levels) might indicate that these patients with severe asthma are suitable for anti-TSLP therapy.

The previous history of severe-to-serious exacerbation is an independent factor predicting future exacerbation (AOR = 3.27). This result was consistent with that of a previous study that recent severe asthma exacerbations are an important independent predictor of future severe exacerbation in children with severe/difficult-to-treat asthma^59^. Similarly, a prospective analysis of patients aged ≥ 12 years with severe/difficult-to-treat asthma indicated that recent severe asthma exacerbations appear to be a strong independent factor predicting future exacerbations (AOR = 3.77)^60^. These findings should prompt physicians to understand the contributing factors and pathological process driving these exacerbations and refine asthma management to prevent future exacerbation.

Our study has several limitations. First, serial examination of serum biomarkers was not performed to delineate the relationship between changes in biomarkers and asthma control status. Second, all study subjects were under maintenance treatment. Multi-treatment might have influenced the levels of the biomarkers at the initiation of the study. Third, this study was observational in nature, and replicating the results in another cohort is needed. Furthermore, whether the strategy to reduce serum TSLP levels, serum tryptase levels, or blood eosinophil counts in these patients with severe asthma can reduce future development of asthma exacerbation remains to be tested. Therefore, further validation must be performed. Nevertheless, the estimated power (1-β) was 0.99 for our sample size.

## Conclusion

We determined that previous history of severe-to-serious exacerbation, blood eosinophil counts, and serum tryptase and TSLP levels were independently associated with the risk of future exacerbation in severe asthma despite receiving multiple therapy. TSLP potentially played the pathogenic role across different asthma phenotypes. TSLP and tryptase levels may be implicated in steroid resistance/responsiveness in the asthma inflammatory process. Low serum tryptase levels and high blood eosinophil counts predict the high risk of future asthma exacerbation. These findings should prompt physicians to understand the contributing factors and pathological process driving these exacerbations and refine asthma management to prevent future exacerbation.

## Supplementary Information

Supplementary Figure S1.

## Data Availability

The data that support the findings of this study can be obtained from the corresponding author upon reasonable request.
