# Supplementary material for: Blood tryptase and thymic stromal lymphopoietin levels predict the risk of exacerbation in severe asthma
Source: Sci Rep. 2021 Apr 19;11:8425. doi: 10.1038/s41598-021-86179-1 (PMC8055991; doi:10.1038/s41598-021-86179-1)

**Blood tryptase and thymic stromal lymphopoietin levels predict the risk of exacerbation in severe asthma**

Hsin-Kuo Ko, Shih-Lung Cheng , Ching-Hsiung Lin, Sheng-Hao Lin , Yi-Han Hsiao, Kang-Cheng Su, Chong-Jen Yu , Hao-Chien Wang , Chau-Chyun Sheu, Kuo-Chin Chiu* , Diahn-Warng Perng *


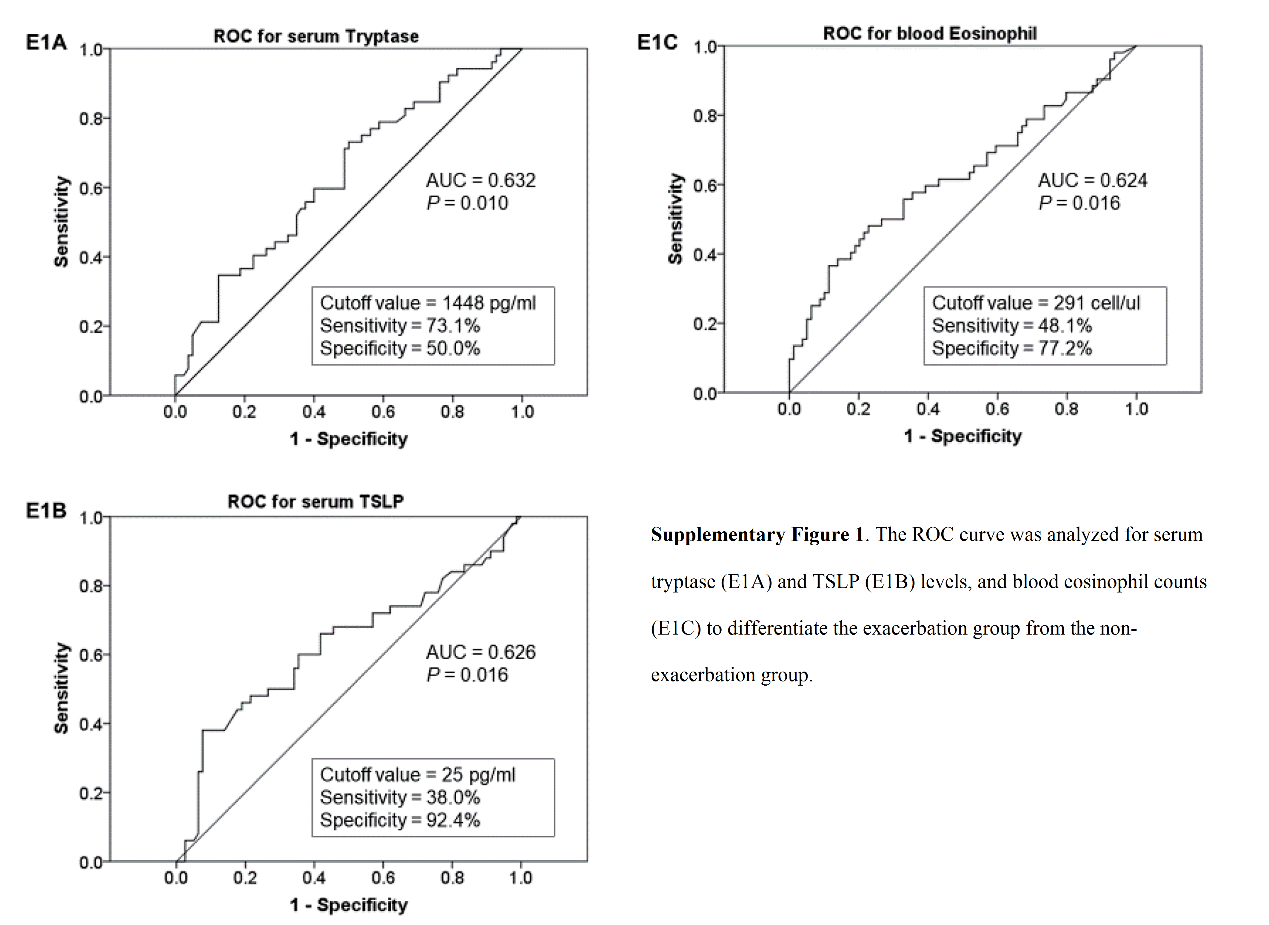

Supplement: Supplementary file 1 — Supplementary Figure S1. [file 41598_2021_86179_MOESM1_ESM.docx]
